# Ecological factors drive natural selection pressure of avian aryl hydrocarbon receptor 1 genotypes

**DOI:** 10.1038/srep27526

**Published:** 2016-06-10

**Authors:** Ji-Hee Hwang, Jin-Young Park, Hae-Jeong Park, Su-Min Bak, Masashi Hirano, Hisato Iwata, Young-Suk Park, Eun-Young Kim

**Affiliations:** 1 Department of Life and Nanopharmaceutical Science and Department of Biology, Kyung Hee University, Hoegi-Dong, Dongdaemun-Gu, Seoul 130-701, Korea; 2 Nature Conservation Research Division, National Institute of Environmental Research, Hwangyoungro 42, Seo-Gu, Incheon 404-708, Korea; 3 Center for Marine Environmental Studies (CMES), Ehime University, Bunkyo-cho 2-5, Matsuyama, 790-8577, Japan

## Abstract

The aryl hydrocarbon receptor (AHR) mediates dioxin toxicities. Several studies have suggested that two amino acid residues corresponding to the 324^th^ and 380^th^ positions in the ligand binding domain (LBD) of the chicken AHR1 (Ile_Ser as high sensitivity, Ile_Ala as moderate sensitivity, and Val_Ala as low sensitivity), could be an important factor determining dioxin sensitivity in avian species. Here, we analyzed the association between ecological factors and AHR1 LBD genotypes of 113 avian species. Cluster analyses showed that 2 major clusters and sub-clusters of the cluster 3 were associated with specific AHR1 genotypes depending on the food, habitat, and migration of the animal. The majority of the species with Ile_Ala type were the Passeriformes, which are omnivorous or herbivorous feeders in the terrestrial environment. The species with Val_Ala type was primarily composed of raptors and waterbirds, which have been exposed to naturally occurring dioxins. An *in vitro* reporter gene assay revealed that the sensitivity to a natural dioxin, 1,3,7-tribromodibenzo-*p*-dioxin was in the order of Ile_Ser > Ile_Ala > Val_Ala. These results suggest that ecological factors related to the exposure of natural dioxins contribute to natural selection of the avian AHR1 genotype, which consequently leads to different sensitivity to man-made dioxins.

Contamination with dioxin-like compounds (DLCs), including polychlorinated dibenzo*-p*-dioxins, polychlorinated dibenzofurans (PCDD/Fs), and coplanar polychlorinated biphenyls (PCBs), are of great environmental concern due to their widespread presence in the ecosystem and high toxicity to humans and wildlife^1^,^2^. After exposure to DLCs, some avian species, such as fish-eating birds, have suffered from reproductive impairment due to a high incidence of embryonic mortality and edema. These species also had developmental abnormalities, including feather loss, crossbill, maldeveloped limbs, and supernumerary digits. As a result of these adverse effects, DLCs caused severe declines of some avian populations in the Great Lake region of North America^3^,^4^,^5^,^6^.

Toxic effects of DLCs are mediated by a ligand-dependent nuclear transcription factor, the aryl hydrocarbon receptor (AHR), which is a member of the basic-Helix-Loop-Helix (bHLH) and Per-Arnt-Sim (PAS) family of proteins. In the absence of a ligand, AHR is stable due to interactions with chaperones, including two molecules of heat shock protein 90 (Hsp90), prostaglandin E synthase3 (p23), and the immunophilin-like protein hepatitis B virus X-associated protein 2 (XAP2) in the cytosol^7^. Upon binding with ligands like DLCs, the AHR relocates to the nucleus to form a heterodimer with its partner molecule, aryl hydrocarbon receptor translocator (ARNT). The ligand-bound AHR eventually transactivates cytochrome P450 1 A (CYP1A) and other genes by binding to a specific dioxin-responsive element, which has a core sequence of 5′-TNGCGGTG-3′ located in the promoter region of these target genes^8^. Induction of CYP1A is thus considered to be an indicator of AHR activation after exposure to DLCs^9^.

The basic molecular mechanism of the AHR-mediated signaling pathway is evolutionarily conserved in avian species as well as mammals. Although mammals have only a single AHR, birds have at least two AHR isoforms, AHR1 and AHR2^10^,^11^. Despite the evolutionary conservation of the AHR-mediated signaling pathway in birds, earlier *in vitro* and *in vivo* studies have reported large interspecies differences in sensitivity to exposure to 2,3,7,8-tetrachlorodibenzo-*p*-dioxin (TCDD) and other DLCs^12^,^13^. Some previous studies have suggested that an *in vitro* assay system constructed using AHR expression vectors from chicken and other avian species may be a valuable tool for evaluating interspecies differences in responses to DLCs, and consequently for assessing risks for the species concerned^14^,^15^,^16^,^17^,^18^.

The varying degrees of TCDD sensitivity in avian species have been explained by sequence differences in the ligand binding domain (LBD) of avian AHR1s, specifically two amino acid residues corresponding to Ile-324 and Ser-380 in the chicken AHR1 (*ck*AHR1)^19^. Three genotypes divergent at the corresponding sites have been found in avian AHR1 orthologs. The AHR1 LBD genotype is classified into high (Ile_Ser), moderate (Ile_Ala), and low sensitivity types (Val_Ala)^13^,^19^. In previous studies, we have shown that the black-footed albatross AHR1 (*bfa*AHR1) has Ile-325 and Ala-381 at the corresponding sites, and the common cormorant AHR1 (*cc*AHR1) has Val-325 and Ala-381^14^,^15^,^16^. The TCDD-EC_50_ values for AHR1-mediated transactivation were in the order of *ck*AHR1 (0.030 nM) < *bfa*AHR1 (0.077 nM) < *cc*AHR1 (0.36 nM) as expected from the AHR1 genotype. Furthermore, *in silico* docking simulations of avian AHR1 and TCDD interactions suggested that the thermodynamic stability of the two amino acid residues involved in the interaction with TCDD reflect the sensitivity to TCDD in these avian species^20^.

It has been reported that sensitivity to DLCs in avian species may be AHR1 dependent, due to the excessive amount of mRNA detected compared to other isoforms^15^,^16^,^21^. This implies that the three AHR1 genotypes have been subjected to natural selection in avian species; however, whether their presence can be completely attributed to natural selection in avian species during evolution and what ecological factors might have contributed to selection are still unknown.

Here, we hypothesize that ecological factors have driven natural selection pressures on AHR1 genotypes in the evolutionary process of avian species, and eventually have led to the interspecies differences in the sensitivity to DLCs. To address these questions, we investigated the amino acid sequences of AHR1 LBDs of 14 Far East avian species. We statistically analyzed the association between ecological factors and avian AHR1 LBD genotypes by the combination of two-way cluster analysis and nonmetric multidimensional scaling (NMDS) in these AHR1 LBD sequences as well as those of 99 avian species deposited in GenBank. In the present study, we explored the ecological factors that may have affected the selection of AHR1 genotypes in avian species.

## Results

### Sequence analysis of avian AHR1 LBD amino acids

The cDNAs of AHR1 LBDs from the blood and liver samples of 14 Far East species were sequenced. Among the 14 species examined, the AHR1 from 13 of the species was classified as a moderately sensitive type (Ile_Ala), with only the grey-headed woodpecker harboring a sequence type associated with low sensitivity (Val_Ala) (Fig. S1). To enhance our sample size, the AHR1 amino acid sequences of 99 additional avian species were obtained from Genbank (Table S1).

We examined a total of 113 species belonging to 21 of the 40 extant avian orders^22^ as classified by the International Ornithological Congress (IOC). The Passeriformes comprised 39.8% of the total species included in the present study. Considering that Passeriformes is the largest order of avifauna, which consists of approximately 50% of the 10,000 bird species known, the species composition of our samples was considered appropriate in order to analyze the selection pressures on AHR1 genotypes by ecological factors.

All of the 113 avian AHR1 genotype profiles were classified into three sensitivity types according to their amino acid sequences (Ile_Ser, Ile_Ala, and Val_Ala types), which correspond to the 324^th^ and 380^th^ amino acid positions in *ck*AHR1. The classification demonstrated that of the 113 species, the highly sensitive Ile_Ser type accounted for 4.4% of amino acid sequences analyzed. Considering that this highly sensitive type was a minority of the avian AHR1 genotypes, birds with this genotype may have a disadvantage when adapting to the environment in comparison to the other genotypes. The Ile_Ala and Val_Ala types accounted for 56.6% and 39.0% of all species, suggesting that these two types are ubiquitous AHR1 genotypes in avian species.

To determine the extent of sequence conservation of the AHR1 LBD among the avian species, CLUSTALW was used to construct pairwise amino acid alignments for each AHR1 genotype (Fig. S2). The percentage identity among AHR1 LBD amino acid sequences was 95~100% in the Ile_Ser type, 93~100% in the Ile_Ala type, and 99~100% in the Val_Ala type. In the alignment of the Ile_Ser type, there were five substitutions at sites corresponding to the 297^th^, 298^th^, 305^th^, 337^th^, and 387^th^ amino acid residues (Fig. S2A). Of these sites, the 297^th^, 298^th^, and 387^th^ amino acid residues comprised a portion of the β-sheet structure, whereas the 305^th^ and 337^th^ were involved in the α-helix (Fig. S3A). In the case of the Val_Ala type, only one mutation was found at the 360^th^ site in the Humboldt penguin AHR1 (Fig. S2B). This amino acid residue forms part of the β-sheet structure, but is not a component of the ligand binding cavity, suggesting that this mutation does not affect sensitivity to DLCs (Fig. S3C). In the Ile_Ala type alignment, five mutations at sites corresponding to the 297^th^, 304^th^, 337^th^, 362^nd^, and 387^th^ amino acids in the homology model of the black-tailed gull AHR1 were identified (Fig. S3B) and an insertion of five amino acids was found in the swan goose AHR1 (Figs S2C and S3D).

### Phylogenetic analysis of avian AHR1 LBD

The phylogenetic tree of AHR1 LBD is divided into two clusters according to genotype (Fig. S4). The first clade includes waterbirds (ducks, herons, cormorants, and gulls) and raptors (owls, hawks, vultures, and ospreys), which mainly harbor the Val_Ala type (species shaded blue in Fig. S4). The second clade is mainly composed of passerines, pheasants, and quails, which have the Ile_Ala type sequence (species shaded yellow in Fig. S4). Although the Ile_Ser type has a scattered distribution in this phylogeny, the major clusters are mainly divided between the Ile_Ala and Val_Ala type. Thus, the results demonstrate that these two amino acids have played a critical role in the evolution of the avian AHR1 LBD.

**Figure 1:**
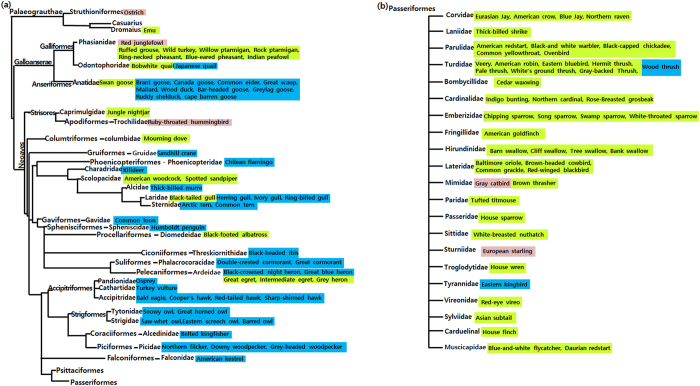
Distribution of AHR1 genotypes of 113 avian species in a phylogenic tree of avian species constructed by Prum *et al*.^23^. Each AHR1 genotype is distinguished by color: pink (highly sensitive Ile_Ser type), green (moderately sensitive Ile_Ala type), and blue (low sensitivity Val_Ala type). (**a**) The distribution of avian species excluding the Passeriformes. The Val_Ala type was favored in 13 orders (Anseriformes, Phoenicopteriformes, Gruiformes, Gaviformes, Ciconiiformes, Suliformes, Sphenisciformes, Charadriiformes, Accipitriformes, Strigformes, Coraciiformes, Piciformes, and Falconiformes). Ile_Ala type was favored in Galliformes. (**b**) The distribution of genotypes in the Passeriformes, the Ile_Ala type was the most common.

To understand the distribution of avian AHR1 genotypes as it relates to the traditional classification of birds, information on AHR1 genotypes of the 113 species was given in the phylogenetic tree constructed by Prum *et al*. (Fig. 1)^23^. The result demonstrates that particular AHR1 genotypes are dominant in certain avifauna groups. The moderately sensitive Ile_Ala type was dominant in the Phasianidae of the Galliformes, the Scolopacidae of the Charadriiformes, and the Passeriformes (species shaded green in Fig. 1). The low sensitivity Val_Ala type was dominant in the Anatidae of the Anseriformes, the Phalacrocoracidae of the Suliformes, the Charadriiformes (excluding the Scolopacidae), the Accipitriformes, the Strigiformes, the Falconiformes, the Coraciiformes, and the Piciformes (species shaded blue in Fig. 1). For the highly sensitive Ile_Ser type, no preference to order was observed (species shaded red in Fig. 1). Thus the acquisition of a specific AHR1 genotype in the species, which are evolutionarily close and share similar ecological conditions, may be advantageous for survival and evolutionary adaptation.

### Composition of ecological factors

To understand the contribution of ecological factors to the acquisition of the avian AHR1 genotype, we analyzed the composition of various ecological factors for each species using a cross tabulation test (Fig. S5). Each AHR1 genotype was composed of different proportions of ecological factors related to habitat (χ^2^ = 60.975, *p* < 0.001) and food types (χ^2^ = 50.625, *p* < 0.001), except for the Ile_Ser type due to its small sample size (4% of all species) (Fig. S5).

For the habitat type, 62% of the Val_Ala type species inhabit the aquatic environment, which includes wetlands, as well as marine coastal, supratidal, and neritic areas (Fig. S5A). The terrestrial environment (forests, shrublands, and grasslands) was a major habitat for the species of both the Ile_Ala and Ile_Ser types, accounting for over 60% of all animals sampled. These were the most distinct results describing species proportions in habitat type distribution according to the AHR1 genotype (χ^2^ = 60.975, *p* < 0.001). For the food type, Val_Ala type species were composed of more various feeding habits than those in other genotype species. Over 50% of Val_Ala species showed carnivorous feeding habit, including invertebrates, vertebrates, and aquatic arthropods (Fig. S5B), while the species with other genotypes were dominantly omnivorous feeders (χ^2^ = 50.625, *p* < 0.001). Concerning the migration type (Fig. S5C), the migratory species were mainly characterized by the Ile_Ala and Val_Ala types. The migratory species in these genotypes were present in similar levels (χ^2^ = 5.449, *p* > 0.05). Analysis of nesting patterns showed no distinct relationship with AHR1 genotypes (χ^2^ = 20.775, *p* > 0.05) (Fig. S5D). Collectively, the cross tabulation analysis demonstrated that habitat and food patterns had significant relationships with the AHR1 genotypes; however, the results from the analysis of the Ile_Ser type data were unreliable due to the small sample size.

### Relationship between ecological factors and AHR1 genotypes

**Figure 2:**
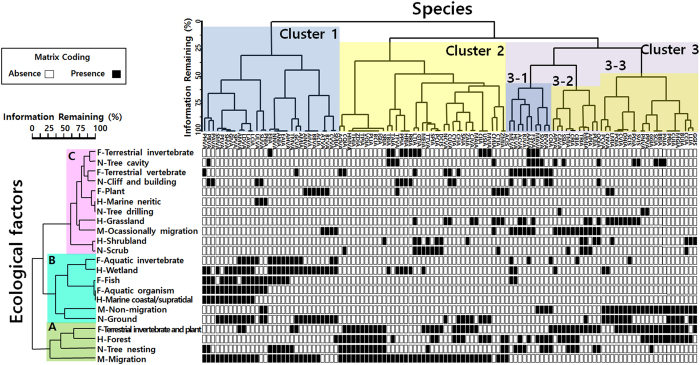
Diagram of two-way cluster analysis. The horizontal matrix axis consists of 113 avian species coded by scientific name (former two letters) and AHR1 genotype (latter two letters), and the vertical axis is the ecological factors. For example, the herring gull (*Larus argentatus*), which has the Val_Ala type, was coded LAVA. In case that the initial of the scientific species name overlapped with others, the second letter of the name was used for coding. The matrix was constructed depending on presence (black) and absence (white) according to ecological factors affecting each species. The branch length indicates the percentage of information that shows the data coverage by ecological factors. A total of 113 species were grouped into 3 clusters (cluster 1, 2, and 3) and 3 subclusters (3-1, 3-2, and 3-3) according to ecological factors. The Val_Ala genotype was dominant in avian species that inhabit marine and wetland ecosystems (cluster 1) or are carnivorous (cluster 3-1). The Ile_Ala type prevailed in avian species that inhabit terrestrial ecosystems (clusters 2, 3-2, and 3-3). Each cluster is shaded according to the dominant genotype (blue for Val_Ala type and yellow for Ile_Ala type). Ecological factors formed 3 clusters (A, B, and C). Cluster A consisted of factors linked to the forest habitat while cluster B was composed of marine and wetland species. The remaining ecological factors were included in cluster C. Ecological factors are indicated according to color, with green, mint, and pink boxes.

The relationships between ecological factors and the AHR1 genotypes were investigated using a two-way cluster analysis and NMDS. In the two-way cluster dendrogram, the ecological factors were classified according to similarities in the configuration of species group (Fig. 2). Cluster A includes the forest habitat and omnivorous feeding type, as well as tree nesting and migration types, with the most represented species from the Passeriformes. Cluster B includes the ecological factors of water birds which are ground-nesting and non-migratory species, and consisted of food types, including invertebrates, fish, and aquatic arthropods, as well as the wetland, marine coastal, and supratidal habitat types. Cluster C was composed of a variety of ecological factors including plant feeding, occasionally migration, and nesting in tree cavities as well as cliffs, buildings and scrub. These clustering patterns demonstrate that ecological factors have high mutual dependency and are closely related to each other.

**Figure 3:**
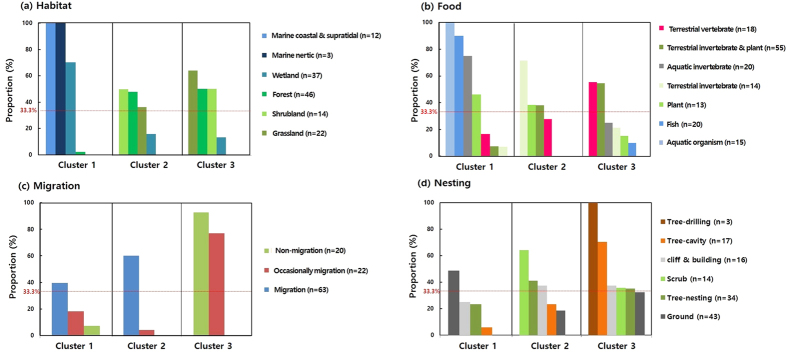
Proportion of ecological factors that contribute to the three clusters. We calculated the proportion of ecological factors by dividing the number of “presence” in a cluster by the total number of “presence” in the matrix coding in Fig. 2. The “presence” number (n) of each ecological factor is noted in the bracket. Major contributing factors in each cluster were selected based on the higher proportion rather than the equal probability that affects the three clusters (33.3%).

**Figure 4:**
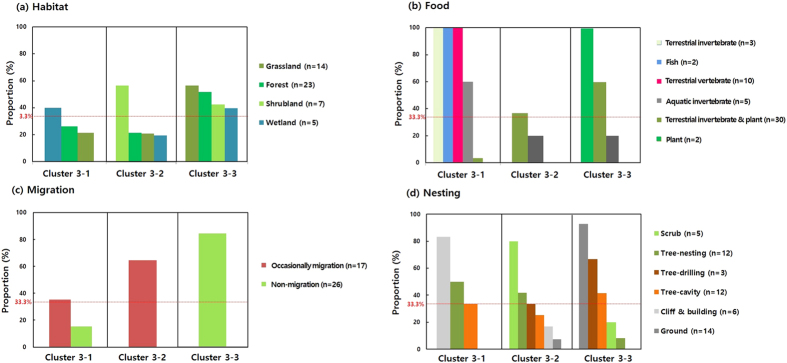
Proportion of ecological factors that contribute to sub-clusters in cluster 3. We calculated the proportion of ecological factors by dividing the number of “presence” in a sub-cluster by the total number of “presence” in the matrix coding in Fig. 2. The “presence” number (n) of each ecological factor is noted in the bracket. Major contributing factors in each sub-cluster were selected based on the higher proportion rather than the equal probability that affects the three sub-clusters (33.3%).

In the two-way cluster analysis, two main clusters (cluster 1 and 2) and three sub-clusters (cluster 3-1, 3-2, and 3-3) of avian species were grouped according to the similarity of ecological factors. We investigated the major factors of each cluster to explain the relationship between the AHR1 genotypes and ecological factors, based on equal probability (Figs 3 and 4). The birds in cluster 1 primarily inhabit wetland or marine environments and feed on fish, aquatic arthropods or plants, thus aquatic habitats are the primary ecological factor for this group. A majority (74%) of the species examined in cluster 1 have the low sensitivity AHR1 genotype (Val_Ala type). Water birds (heron, duck, goose, cormorant, gull, and penguin) and a piscivorous raptor, the osprey, are also a part of this cluster (Table 1). Avian species from cluster 2, 3-2, and 3-3 inhabit inland regions including forests, shrublands, and grasslands. The migratory type is the main factor accounting for the differential grouping of cluster 2 and 3. Cluster 2 mainly consists of migratory birds, which inhabit forests or shrublands and are omnivorous, insectivorous, and herbivorous species. The moderately sensitive Ile_Ala AHR1 genotype was found in 72% of the species in this cluster that belong to the passerine group (Table 1). Cluster 3 consists of non-migratory or short-distance migratory birds and is further divided into 3 sub-clusters. Compared to the other groups, sub-cluster 3-1 is composed of the species with the most variable food types, including terrestrial invertebrates, terrestrial vertebrates, fish, and aquatic invertebrates. The animals in this group, including raptors, kingfishers, and crows (Table 1), share vertebrates as a common food type and 90% of them have the Val_Ala type AHR1. Sub-cluster 3-2 contains omnivorous species that inhabit shrubland and occasionally migrate, with a total of 82% of these species sharing the Ile_Ala type sequence (Table 1). Additionally the passerines, which occasionally migrate, are also part of this sub-cluster. The species of sub-cluster 3-3 are omnivorous and non-migratory and include pheasants, quails, and non-migratory passerines which are mainly composed of Ile_Ala (68%) genotype representatives. Interestingly, the proportion of species with the Ile_Ser type was higher in this cluster than in the others (Fig. 2 and Table 1).

**Figure 5:**
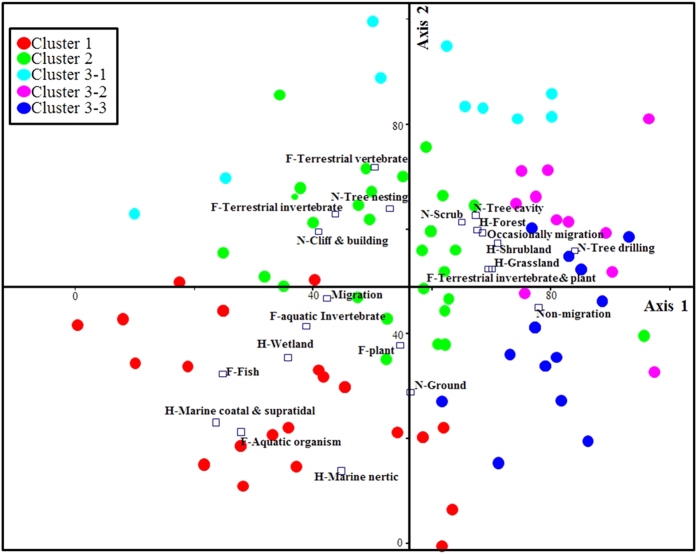
Data distribution obtained by NMDS. Ecological factors were marked with an initial (H: habitat, F: food, M: migration, N: nesting type). Each colored circle represents the clusters that each species belongs to. An open square represents the coordinate of each ecological factor in the NMDS graph. The distribution of avian species along two axes after the NMDS analysis was consistent with the results from the of two-way cluster analysis. Marine and wetland ecosystem factors separated species of cluster 1 from others, and terrestrial species were divided into 4 clusters according to 4 categories of ecological factors.

In order to further verify our results, the NMDS was conducted on the ecological factor and avian AHR1 genotype datasets. The data accounted for 79% of the distribution along axis 1 and 2 and was statistically significant (*p* < 0.05). Each colored circle in Fig. 5 represents the clusters to which each species belongs as shown by the cluster analysis. Species in cluster 1 were distinguished by marine and wetland ecosystem factors, and the terrestrial species were divided into three clusters according to 4 ecological factor categories (Table S2). Thus data distribution along the two axes of the NMDS were consistent with the results of two-way cluster analysis (Fig. 5).

### Response of AHR1 genotypes to a naturally occurring dioxin

**Figure 6:**
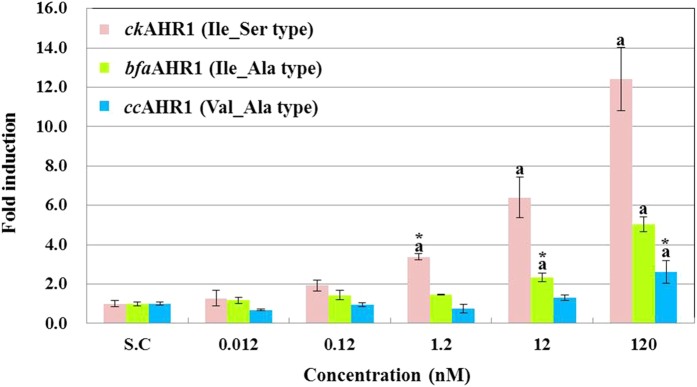
Comparison of transactivation potencies of avian AHR1 Ile_Ser type (*ck*AHR1), Ile_Ala type (*bfa*AHR1), and Val_Ala type (*cc*AHR1) by exposure to a naturally occurring dioxin, 1,3,7-TriBDD in *in vitro* reporter gene assay. The RLU values in all wells were normalized by the mean of RLU values in solvent control (S.C.) wells. All data were shown as mean ± standard deviation (SD). (**a**) significant change compared with RLU values in S.C. *LOEC.

This study reveals that certain avifauna groups favor particular AHR1 genotypes from phylogenetic and ecological factor analyses. In particular, waterbirds and raptors with a low sensitive Val_Ala AHR1 genotype might have a selective advantage under conditions that the high exposure level to naturally occurring dioxins continuously takes place in the environment; the exposure to these natural dioxins may be a natural selection pressure of the AHR1 genotype in avian species. To examine this assumption, we compared the transactivation potencies of three AHR1 genotypes (*ck*AHR1: Ile_Ser type, *bfa*AHR1: Ile_Ala type, and *cc*AHR1: Val_Ala type) by the exposure to a naturally occurring dioxin, 1,3,7-tribromodibenzo-*p*-dioxin (1,3,7-TriBDD) in *in vitro* reporter gene assay. The result showed that the Ile_Ser type had the lowest LOEC value (1.2 nM), followed by Ile_Ala type (12 nM) and Val_Ala type (120 nM) (Fig. 6).

## Discussion

We initially compared the amino acid sequences of AHR1 LBDs of 113 avian species. The results showed that only a few mutations were identified between the species with the same AHR genotypes. An earlier study focused on site-directed mutagenesis of avian AHR1s demonstrated that the Ile-324 and Ser-380 of *ck*AHR1, and Val-325 and Ala-381 of the common tern AHR1 are critical sites involved in the binding of TCDD to AHR1^19^. An *in vitro* chimeric AHR1 reporter gene assay proved that four of the variable sites, other than Ile/Val-324/325 and Ser/Ala-380/381, do not change the transactivation potency of AHR1 by dioxin exposure^24^. Furthermore, our *in silico* simulation of the molecular dynamics of AHR1 also supports the assumption that these two amino acids are critical in the interaction with TCDD^20^. In case of the five insertions in the sequences of swan goose AHR1, the insertions are not located in the region that interacts with TCDD^20^; therefore, they should also not affect the sensitivity to DLCs in the species harboring Ile_Ala type sequences. Collectively, AHR1-mediated dioxin sensitivity can be determined by sites corresponding to 324^th^ and 380^th^ amino acid residues of *ck*AHR1, including the Ile_Ser, Ile_Ala, and Val_Ala types.

Recently, involvement of AHR in physiology such as immune system maintenance, protein degradation, and cell proliferation has been elucidated^25^,^26^. In adaptive immunity, AHR plays a pivotal role in anti-bacterial defense^27^ and disease tolerance to xenobiotics^28^,^29^. Due to the promiscuous nature of the binding pocket, AHR can be activated by various ligands not only polycyclic aromatic hydrocarbons (PAHs) and DLCs but also those that are naturally derived, including tryptophan metabolites^30^ and bacterial pigments^27^. FICZ (6-formylindolo [3,2-b] carbazole) is a tryptophan metabolite and has a high affinity to AHR^31^,^32^. Recent studies have shown that transactivation potencies of FICZ are greater than TCDD regardless of the avian AHR1 genotype^20^,^33^. In addition, there are fewer inter-species differences in AHR1 sensitivity to this endogenous ligand^33^, whereas there are large differences in responses to TCDD of the AHR1 among species^20^,^24^. Our previous study also supported that there was no linkage between avian AHR1 genotypes and AHR1-mediated responses to FICZ^34^. This implies that the exposure to naturally occurring dioxins under various ecological conditions has exerted selection pressure on the AHR1 genotype with different dioxin sensitivity in the evolutionary process of avian species. To prove this assumption, we analyzed the relationship between ecological factors and avian AHR1 genotypes.

An earlier study demonstrated that the genotype distribution of key amino acids, corresponding to the 324th and 380th amino acid positions of *ck*AHR1, in avian AHR isoforms could not be predicted from the phylogenetic tree or previously constructed taxonomy^35^. On the other hand, when we investigated the genotype distribution using our larger data set of 113 avian species, certain AHR1 genotypes were dominant in the Neoaves. The majority of the passerine have a moderately sensitive Ile_Ala AHR1 genotype, whereas waterbirds and raptors have a less sensitive Val_Ala AHR1 genotype. The species with the Val_Ala AHR1 genotype are in high trophic positions of the food web, which feed on fish, invertebrates, and vertebrates, and could have been exposed to high levels of various naturally synthesized dioxins. The species with a moderately sensitive Ile_Ala AHR1 genotype tend to be omnivorous feeders and mainly inhabit terrestrial ecosystems (Fig. 2). Since most passerines live on land, the terrestrial ecosystem is a major contributing factor to the preference for this genotype.

When we compare the previously reported sequences of various vertebrates including fish, birds and mammals, Ile_Ala type is the most common genotype. Therefore we assume that Ile_Ala type may be a primary genotype in vertebrates. Recent studies have reported that natural sources of dioxins exist in the terrestrial^36^ and aquatic ecosystem^37^. Naturally occurring dioxins in terrestrial ecosystem included 1,3,6,8- and, 1,3,7,9-tetrachlorodibenzo-*p*-dioxins, and 2,4,6,8-tetrachlorodibenzofuran, of which the congener profile differs from that of anthropogenic sources^38^. These congeners are synthesized when 2,4-dichlorophenol is allowed to react with the fungal enzyme chloroperoxidase in the slime mold, *Dictyostelium purpureum*^39^, and lichens, *Lecanora cinereocarnea*^40^ and *Lecanora iseana*^41^. In the marine ecosystem, notable concentrations of polybrominated dibenzo-*p*-dioxins (PBDDs) including 1,3,7-TriBDD have been detected in Baltic Sea biota^37^. It has been suggested that some PBDD congeners are synthesized in red algae by UV irradiation of hydroxylated polyborminated dipheyl ethers (OH-BDE)^42^. These naturally synthesized dioxins could be assimilated by filter feeders like mussels, and then transferred to mussel-eating fish, e.g., perch^37^,^43^. Given these findings, it is possible that raptors and waterbirds have been exposed to high levels of naturally occurring dioxins in the process of evolutionary adaptation. Thus this might be a driving force to differentiate AHR1 genotypes. In this study, we examined this hypothesis by using an *in vitro* reporter gene assay where each of three AHR1 genotypes was expressed in COS-7 cells treated with a natural dioxin, 1,3,7-TriBDD. The results revealed that the sensitivity to the natural dioxin was in the order of Ile_Ser > Ile_Ala > Val_Ala as expected (Fig. 6). Therefore, the acquisition of Val_Ala AHR1 genotype in waterbirds and raptors might be due to the high exposure levels of natural dioxins to mitigate the toxicity of natural dioxins. The highly sensitive Ile_Ser AHR1 genotype was found only in five species, implying that this type is rare in birds. Although it is still unclear how this genotype affects avian adaptation, its rarity suggests that it could be disadvantageous or perhaps impart a selective competition advantage to a few selected species.

Previous studies have found that the function of specific genes can be altered according to the environment, as is the case with heat shock proteins^44^. It is well known that the aggregate or high constitutive expression level of heat shock protein 70 in thermophilic species leads to its heat resistance^45^. Likewise, an AHR1 genotype in avian species might be preferred in a certain common environment such as the exposure to naturally occurring dioxins, and this could be beneficial to their fitness. Here, we hypothesized that differential sensitivity to DLCs in avian species may be due to various AHR1 genotypes that have evolved under unique environmental circumstances. In conclusion, this study suggests that the exposure to natural dioxins could have enforced natural selection pressure on avian AHR1 genotypes, which consequently led to different sensitivities to man-made dioxins.

## Materials and Methods

### Collection of avian AHR1 LBD amino acid sequences

The AHR1 LBD amino acid sequences of 99 avian species reported in previous studies were obtained from the GenBank database (Table S1). Since these AHR1 LBDs mostly belong to North American species, we additionally sequenced 14 Far-East avian AHR1s in this study. The sequencing method for the Far-East species was described in the supporting information. In order to compare the AHR1 amino acid sequences from individual species, we conducted CLUSTALW alignment. Pairwise alignments were made to evaluate the amino acid identities among AHR1s using Mac Vector 7.1.

### Phylogenetic analysis

To clarify the evolutionary relationship of AHR1 from each species, we conducted a phylogenetic analysis of AHR1 LBD nucleotide sequences using BEAUti and BEAST 1.7 (Bayesian evolutionary analysis sampling trees)^46^. To understand the distribution pattern of avian AHR1 LBD genotypes in the evolutionary classification of birds, we cited the latest avian phylogenetic tree provided by Prum *et al*.^23^ and highlighted the AHR1 genotypes in the tree.

### Data collection of ecological factors affecting 113 avian species

The data on the ecological factors of each avian species were compiled from “Birds of Korea”^47^ and “All about birds” from the lab of Ornithology at Cornell University. The collected data were categorized as one of four ecological factors: habitat, food, nesting, and migration type (Table S2). We followed the criteria of IUCN red list classification schemes to classify the habitat (http://www.iucnredlist.org/). For the wetland category, we combined wetland, artificial aquatic, and marine intertidal areas, in accordance with the definition of wetland reported by Ramsar Convention Secretariat^48^, which includes marshes, peat lands, floodplains, rivers, lakes, and coastal areas. The avian diet was classified according to food sources that are the most representative in each species. The “aquatic arthropods” was defined as all aquatic organisms except fish and aquatic invertebrates, and an omnivorous diet as consisting of “terrestrial invertebrates and plants” for the species that consume insects in the breeding season and feed on plant-derived materials thereafter. Regarding migration types, we added “occasional migration” as the type of migration for the species that demonstrate a mix of behaviors, including residence and short-distance migration. In addition, we defined types of nesting related to tree according to the differences in the behavioral ecology of nesting for precise analysis of ecological factors: (1) tree nesting type, which builds nests on the branch, (2) tree cavity type, which uses the cavity on the tree not constructed by birds, and (3) tree drilling type, which uses the cavity drilled by birds. Details concerning each ecological factor used for the analysis are listed in Table S2.

### Measurement of responses of avian AHR1s to 1,3,7-TriBDD

To compare the response of avian AHR1 genotypes to a naturally occurring dioxin, 1,3,7-TriBDD, we conducted an *in vitro* luciferase reporter-gene assay using cDNA clones of Ile_Ser type, chicken AHR1 (*ck*AHR1), Ile_Ala type, black-footed albatross AHR1 (*bfa*AHR1) and Val_Ala type, common cormorant AHR1 (*cc*AHR1).

#### Plasmids

The firefly luciferase vector containing chicken CYP1A5 (*ck*CYP1A5) promoter region, pGL4-*ck*CYP1A5-6XRE was constructed in our previous study^14^. *Renilla* luciferase reporter vector (pGL4.74 [pRL/TK], Promega) was used as a transfection control. The expression vectors of *ck*AHR1, *bfa*AHR1, *cc*AHR1, and chicken ARNT1 (*ck*ARNT1) were previously constructed by inserting the respective full-length cDNAs into pcDNA3.1 zeo (+) (Invitrogen)^10^,^14^,^49^. The total amount of transfected DNA per well was kept constant at 300 ng by the addition of pcDNA3.1/Zero (+) vector without insert.

#### Preparation of standard solutions of 1,3,7-TriBDD

A standard solution of 1,3,7-Tribromodibenzo-*p*-dioxin (1,3,7-TriBDD) dissolved in toluene was purchased from AccuStandard, Inc. (New Haven, CT, USA), and dimethyl sulfoxide (DMSO) was purchased from Sigma-Aldrich (St. Louis, MO). 1,3,7-TriBDD was concentrated under a stream of nitrogen to dryness and dissolved in hexane. DMSO was then added and mixed by a vortex. The hexane layer was dried by rotor evaporation and then by nitrogen flow, afterwards prepared in DMSO as stock solution. The stock solution of 1,3,7-TriBDD was diluted serially (0.012~120 nM) with DMSO for ligand treatment. The final concentration of DMSO was adjusted to 0.1%.

#### *In vitro* AHR transactivation assay

The African green monkey kidney COS-7 cells, which express a little endogenous AHR protein were maintained in Rosewell Park Memorial Institute (RPMI-1640) Medium (Hyclone) supplemented with 10% fetal bovine serum (Hyclone) at 37 °C and 5% CO_2_. Reporter gene assay experiment was performed following the method of Lee *et al*.^21^. Cells (6 × 10^4^ per well) were seeded in 24 well plates (1.9 cm^2^/well). The amounts of transfected vectors were as follows; 5 ng of *ck*AHR1, *cc*AHR1, 50 ng of *ck*ARNT1 expression vectors, 20 ng of pGL4-*ck*CYP1A5 and 5 ng of pGL4.74 control vectors. Transfections of vectors with Lipofectamine LTX (Invitrogen) were carried out 18 h after the seeding of cells. For each well, the plasmid DNAs were mixed with 1 μl of Lipofectamine LTX Transfection Reagent. The mixture was added to cells in serum-free Opti-MEM (Invitrogen). After 5 h incubation, cells were treated for 18 h with the solution of either 0.1% DMSO (control) or 1,3,7-TriBDD, which was diluted in charcoal/dextran-treated RPMI 1640 with 10% charcoal/dextran-treated FBS. The luciferase activity was determined with the Dual Luciferase Assay System (Promega). Final luminescence values were expressed as a ratio of firefly luciferase unit to *Renilla* luciferase (relative luciferase unit, RLU).

### Statistical analysis

#### Statistical analysis for ecological factors

All data were coded using the presence or absence marking method according to the ecological factors identified for each species. The ecological factors that did not belong to the variables defined in the analysis, were coded as “absence”; e.g., the Chilean flamingo, which feeds on algae, and the gray catbird, which needs no nest because it uses brood parasitism as a reproductive behavior, were coded as “absence” for food and nesting types, respectively.

The distributions of ecological factors according to AHR1 genotypes were analyzed with the cross tabulation test to determine the relatedness between certain ecological factors and AHR1 genotypes. When a species belongs to multiple coding categories, the species was coded with all of them. We then conducted a two-way cluster analysis to explore the relationship between the AHR1 genotypes and ecological factors. The distance between data was calculated by using the Euclidean algorithm, and hierarchical clustering was performed by Ward’s method. We then carried out NMDS to explore the ecological factors that may have driven natural selection pressures on the AHR1 genotypes in the evolutionary process of avian species.

A two-way cluster analysis and NMDS was conducted using PC-ORD 5.31 (MjM software design) and the cross tabulation test was performed using SPSS 18.0 (SPSS, Chicago, IL, U.S.A).

#### Statistical analysis for *in vitro* AHR1-mediated responses to 1,3,7-TriBDD

Responses to each concentration of 1,3,7-TriBDD were obtained from at least three replicates in four independent experiments. The RLU values in all wells were normalized by the mean of RLU values in solvent control (DMSO) wells. All data were shown as mean ± standard deviation (SD). The lowest observed effect concentration (LOEC) and the fold change in luciferase induction were determined by ANOVA test using Bonferroni as post-hoc comparisons (*p* < 0.01). ANOVA test was performed by SPSS 18.0 (SPSS, Chicago, IL, USA).

## Additional Information

**How to cite this article**: Hwang, J.-H. *et al*. Ecological factors drive natural selection pressure of avian aryl hydrocarbon receptor 1 genotypes. *Sci. Rep*. **6**, 27526; doi: 10.1038/srep27526 (2016).

## Supplementary Material

Supplementary Information

## Figures and Tables

**Table 1:** Representative ecological factors, species, and AHR1 genotypes in each cluster selected by two-way cluster analysis.

Cluster	AHR1 genotype	Habitat type	Food type	Migration type	Nesting type	Representative species
Cluster 1	Val_Ala type (74%) Ile_Ala type (26%)	Marine, Wetland	Aquatic arthropods, Fish, Aquatic invertebrate	Migration	Ground	Great cormorant, Bar-headed goose
Cluster 2	Ile_Ala type (77%) Val_Ala type (18%) Ile_Ser type (5%)	Shrubland, Forest	Terrestrial invertebrate, Plant, Terrestrial invertebrate and plant	Migration	Scrub, Tree nesting	Pale thrush
Cluster 3-1	Val_Ala type (90%) Ile_Ala type (10%)	Forest	Terrestrial vertebrate, Fish, Terrestrial invertebrate, Aquatic invertebrate	Occasionally migration	Cliff & building, Tree nesting Tree cavity	Bald eagle, Belted kingfisher, Northern raven
Cluster 3-2	Ile_Ala type (82%) Val_Ala type (18)	Forest, Shrubland	Terrestrial invertebrate and plant	Occasionally migration	Scrub, Tree nesting	American crow, Brown thrasher
Cluster 3-3	Ile_Ala type (68%) Val_Ala type (18%) Ile_Ser type (14%)	Forest, Grassland	Plant, Terrestrial invertebrate and plant	Non-migration	Ground, Tree cavity	Blue-eared pheasant, House sparrow
