# Supplementary material for: Ecological factors drive natural selection pressure of avian aryl hydrocarbon receptor 1 genotypes
Source: Sci Rep. 2016 Jun 10;6:27526. doi: 10.1038/srep27526 (PMC4901312; doi:10.1038/srep27526)
Supplement: Supplementary Information [file srep27526-s1.doc]

**Ecological factors drive natural SELECTION pressure of AVIAN ARYL HYDROCARBON RECEPTOR 1 GENOTYPES**

Ji-Hee Hwang^1^, Jin-Young Park^2^*, Hae-Jeong Park^1^, Su-Min Bak^1^, Masashi Hirano^3^, Hisato Iwata^3^, Young-Suk Park^1^*, Eun-Young Kim^1^*

**Supporting Information**

**1. cDNA cloning of AHR1 LBDs from 14 Far East avian species**

Tissue and blood samples were stored in RNAlater (Qiagen, GmbH, Hilden, German) and all 14 Far-East avian species samples were provided by the National Institute of Environmental Research, Korea. To clone AHR1 LBDs from these avian species, total RNA was extracted from liver or blood samples. For total RNA extraction from liver and blood samples, Hybrid-R™ Kit (Gene all, Seoul, South Korea) and SV Total RNA Isolation System Kit (Promega, Madison, U.S.A) were used, respectively. From the extracted total RNA, we synthesized cDNA using iScript cDNA Synthesis Kit (Bio-Rad, California, USA) with a random hexamer under the following conditions: 25°C 5 min, 42°C 30 min and 85°C 5 min. AHR1 LBD cDNA was amplified from using the cDNA library as a template with the forward (5’-CA GAC CAA CTT CCT CCA GAG-3’) and reverse primers (5’-CGC TGC TTG CTG GAT AAC-3’). The amplification of AHR1 LBD was conducted with Amplitaq DNA Polymerase (Applied Biosystems, Foster City, CA) under following conditions: initial denaturation 95°C 1 min 45 s, 27 cyles of 95°C 30 s, 52°C 30 s and 68°C 45 s, followed by final extension at 68°C 7 min. Amplified products were sub-cloned into the pLUG-Prime® T vector (Intron Biotechnology, Seoul, Korea) for sequencing analysis. Sequence analyses of nucleotide and amino acid residues were conducted by MacVector 7.1.

**Table S1. Genbank accession numbers of avian AHR1s used for the statistical analyses in this study. (asterisks: sequenced in the present study)**

| **Name** | **Scientific name** | **Accession no.** | **Name** | **Scientific name** | **Accession no.** |
| --- | --- | --- | --- | --- | --- |
| Red jungle fowl | *Gallus gallus* | NC006089 | European starling | *Sturnus vulgaris* | FJ376538 |
| Gray catbird | *Dumetella carolinensis* | FJ376509 | Ostrich | *Struthio camelus* | AB820092 |
| Ruffed grouse | *Bonasa umbellus* | FJ376525 | Wild turkey | *Meleagris gallopavo* | EU660874 |
| American redstart | *Setophaga ruticilla* | JQ814789 | Baltimore oriole | *Icterus galbula* | FJ376487 |
| Black-capped chickadee | *Poecile atricapilla* | FJ376492 | Brown-headed cowbird | *Molothrus ater* | FJ376495 |
| Chipping sparrow | *Spizella passerina* | FJ376498 | Common grackle | *Quiscalus quiscula* | FJ376501 |
| Indigo bunting | *Passerina cyanea* | FJ376513 | Northern cardinal | *Cardinalis cardinalis* | FJ376516 |
| Red-winged blackbird | *Agelaius phoeniceus* | FJ376521 | Rose-breasted grosbeak | *Pheucticus ludovicianus* | FJ376524 |
| Swamp sparrow | *Melospiza georgiana* | FJ376528 | Tufted titmouse | *Baeolophus bicolor* | FJ376536 |
| Rock ptarmigan | *Lagopus muta* | FJ376523 | American woodcock | *Scolopax minor* | JF969754 |
| American crow | *Corvus brachyrhynchos* | JQ814788 | American goldfinch | *Carduelis tristis* | FJ376484 |
| Bank swallow | *Riparia riparia* | FJ376488 | Barn swallow | *Hirundo rustica* | FJ376534 |
| American cliff swallow | *Petrochelidon pyrrhonota* | FJ376499 | Eastern bluebird | *Sialia sialis* | EU660870 |
| House finch | *Carpodacus mexicanus* | FJ376510 | House sparrow | *Passer domesticus* | FJ376511 |
| Northern raven | *Corvus corax* | JQ969022 | Red-eyed vireo | *Vireo olivaceus* | FJ376519 |
| Veery | *Catharus fuscescens* | JQ814793 | White-breasted nuthatch | *Sitta carolinensis* | FJ376531 |
| Brown thrasher | *Toxostoma rufum* | JQ814790 | Emu | *Dromaius novaehollandiae* | JF950300 |
| Bobwhite quail | *Colinus virginianus* | FJ376494 | Blue-eared pheasant | *Crossoptilon auritum* | AB820094, AB820095 |

**Table S1. Continued**

| **Name** | **Scientific name** | **Accession no.** | **Name** | **Scientific name** | **Accession no.** |
| --- | --- | --- | --- | --- | --- |
| Ruby-throated hummingbird | *Archilochus colubris* | FJ376541 | Pale thrush* | *Turdus pallidus* | KT006866 |
| Ring-necked pheasant | *Phasianus colchicus* | EU660873 | Indian peafowl | *Pavo cristatus* | AB820092 |
| Willow ptarmigan | *Lagopus lagopus* | FJ376532 | Eurasian jay* | *Garrulus glandarius* | KT006864 |
| Black-and-white warbler | *Mniotilta varia* | FJ376491 | Intermediate egret* | *Egretta intermedia* | KT006863 |
| Cedar waxwing | *Bombycilla cedrorum* | FJ376497 | Japanese quail | *Coturnix japonica* | EU660871 |
| Common yellowthroat | *Geothlypis trichas* | FJ376503 | Arctic tern | *Sterna paradisaea* | HQ317441 |
| Ovenbird | *Seiurus aurocapilla* | FJ376518 | Belted kingfisher | *Megaceryle alcyon* | FJ376490 |
| Song sparrow | *Melospiza melodia* | JQ824841 | Common tern | *Sterna hirundo* | AF192503 |
| White-throated sparrow | *Zonotrichia albicollis* | JQ814794 | Downy woodpecker | *Picoides pubescens* | FJ376504 |
| Spotted sandpiper | *Actitis macularius* | FJ376527 | Grey-headed woodpecker* | *Picus canus* | KT006856 |
| American robin | *Turdus migratorius* | FJ376485 | Great horned owl | *Bubo virginianus* | FJ376507 |
| Blue jay | *Cyanocitta cristata* | FJ376493 | Killdeer | *Charadrius vociferus* | FJ376514 |
| Hermit thrush | *Catharus guttatus* | JQ814792 | Ring-billed gull | *Larus delawarensis* | FJ376522 |
| House wren | *Troglodytes aedon* | FJ376512 | Eastern screech owl | *Megascops asio* | FJ376526 |
| Tree swallow | *Tachycineta bicolor* | FJ376530 | Turkey vulture | *Cathartes aura* | FJ376537 |
| Black-footed albatross | *Phoebastria nigripes* | AB106109 | Common eider | *Somateria mollissima* | EU660868 |
| Mourning dove | *Zenaida macroura* | FJ376515 | Wood duck | *Aix sponsa* | EU660875 |
| Swan goose | *Anser cygnoides* | AB820099 | Greylag Goose | *Anser Anser* | AB820103 |
| Blue-and-white flycatcher* | *Cyanoptila cyanomelana* | KT006858 | Black-headed ibis | *Threskiornis melanocephalus* | AB820112 |
| Black-crowned night heron | *Nycticorax nycticorax* | AB820118, AB8200119 | Daurian redstart* | *Phoenicurus auroreus* | KT006859 |

**Table S1. Continued**

| **Name** | **Scientific name** | **Accession no.** | **Name** | **Scientific name** | **Accession no.** |
| --- | --- | --- | --- | --- | --- |
| Scaly thrush* | *Zoothera dauma* | KT006869 | Grey-backed thrush* | *Turdus hortulorum* | KT006861 |
| Thick-billed shrike* | *Lanius tigrinus* | KT006868 | Asian subtail* | *Urosphena squameiceps* | KT006867 |
| Jungle nightjar* | *Caprimulgus indicus* | KT006865 | Great Egret* | *Egretta alba* | KT006862 |
| Grey heron* | *Ardea cinerea* | KT006860 | Black-tailed gull* | *Larus crassirostris* | KT006857 |
| Great blue heron | *Ardea herodias* | FJ376506 | American kestrel | *Falco sparverius* | EU660871 |
| Bald eagle | *Haliaeetus leucocephalus* | FJ376486 | Barred owl | *Strix varia* | FJ376489 |
| Northern flicker | *Colaptes auratus* | FJ376500 | Common loon | *Gavia immer* | FJ376502 |
| Cooper's hawk | *Accipiter cooperii* | JQ814791 | Double-crested cormorant | *Phalacrocorax auritus* | EU660869 |
| Eastern kingbird | *Tyrannus tyrannus* | FJ376505 | Great cormorant | *Phalacrocorax carbo* | AB109545 |
| Herring gull | *Larus argentatus* | DQ371287 | Ivory gull | *Pagophila eburnea* | FJ376540 |
| Osprey | *Pandion haliaetus* | FJ376517 | Red-tailed hawk | *Buteo jamaicensis* | FJ376520 |
| Sandhill crane | *Grus canadensis* | FJ376535 | Northern saw-whet owl | *Aegolius acadicus* | JQ969021 |
| Sharp-shinned hawk | *Accipiter striatus* | JQ969020 | Thick-billed murre | *Uria lomvia* | FJ376529 |
| Brant goose | *Branta bernicla* | FJ376539 | Canada goose | *Branta canadensis* | FJ376496 |
| Greater scaup | *Aythya marila* | FJ376508 | Mallard | *Anas platyrhynchos* | EU660872 |
| Wood thrush | *Hylocichla mustelina* | FJ376533 | Bar-headed goose | *Anser indicus* | AB820101 |
| Ruddy shelduck | *Tadorna ferruginea* | AB820108 | Cape barren goose | *Cereopsis novaehollandiae* | AB820110 |
| Humboldt penguin | *Spheniscus humboldti* | AB820113 | Chilean flamingo | *Phoeniconaias chilensis* | AB820115 |
| Snowy owl | *Nyctea scandiaca* | AB820121 |  |  |  |

**Table S2. Four categories (habitat, food, nesting and migration type) of ecological factors applied for statistical analyses in this study.**

| Ecological factors | Details of ecological factors |
| --- | --- |
| Habitat | Forest, Shrubland, Grassland, Wetland (wetland, artificial aquatic, marine intertidal) , Marine coastal/supratidal, Marine neritic, |
| Food | Plant, Terrestrial invertebrate and plant, Terrestrial invertebrate, Fish, Aquatic arthropods, Aquatic invertebrate, Terrestrial vertebrate |
| Nesting | Tree nesting, Tree cavity, Tree drilling, Scrub, Cliff and building, Ground |
| Migration | Migration, Non-migration, Occasionally migration |

**Figure legends**

Fig. S1- Alignment of nucleotide sequences from 14 Far-East avian AHR1 LBDs. 13 species are the Ile_Ala type, only one species, the grey-headed woodpecker marked with asterisk is the Val_Ala type.

Fig. S2-Alignment of avian AHR1 LBD amino acid sequences categorized by each genotype, Ile_Ser (a), Val_Ala (b) and Ile_Ala (c). Two critical amino acids, corresponding to 324^th^ and 380^th^ in *ck*AHR1, which are critical for the sensitivity to DLCs, are marked with lines. All the amino acid positons are marked based on the red jungle fowl (the chicken). Amino acids, which showed low conservation levels between the species are marked with boxes. (Red asterisks: amino acids which are used for assigning avian AHR1 genotype, Black asterisks: amino acids which showed low levels of conservation in each AHR1 genotype.)

Fig. S3- Homology models of AHR1 LBDs constructed by using Molecular Operating Environment (ver. 2014.09; Chemical Computing Group Inc., Montreal, QB, Canada). (a) ostrich AHR1 (Ile_Ser type) (b) black-tailed gull AHR1 (Ile_Ala type) (c) Humboldt penguin AHR1 (Val_Ala type) (d) swan goose AHR1 (Ile_Ala type). The structures consist of the following: α–helix (red), β-sheet (yellow), loop (grey ribbon) and turn (blue). Pocket volume (Å^3^) of each AHR1 LBD is shown.

Fig. S4- Phylogenic tree based on the nucleotide sequences of avian AHR1 LBDs. The tree was constructed with BEAUti, BEAST 1.7 (Bayesian evolutionary analysis sampling trees) and Figtree v1.4.2. Node labels were displayed by node age. The major clusters appeared to be classified into two AHR1 genotypes: Ile_Ala and Val_Ala types. Blue shading indicates that the Val_Ala type is dominant, which includes water birds (herons, cormorants, and gulls) and raptors (owls, hawks, vultures, and ospreys). Yellow shading indicates that the Ile_Ala type is dominant which is composed of passerines, pheasants, and quails. Distribution of species with the Ile_Ser type is scattered in this tree.

Fig. S5**-** Patterns of species with specific ecological factors in each AHR1 genotype analyzed by cross-tabulation test. (a) Patterns of habitat demonstrate that 62% of Val_Ala type species inhabit the aquatic environment including wetlands and marine ecosystems, and over 60% of the Ile_Ala type species inhabit the terrestrial environment (χ^2^=60.975, p<0.001). (b) As for the pattern of food type, the Val_Ala type species showed more various feeding habits than species from other genotypes. Omnivorous feeders were dominant in the Ile_Ala and Ile_Ser types (χ^2^=50.625, *p*<0.001). (c) Patterns of migration type showed no significant difference between genotypes (χ^2^=5.449, *p*>0.05). (d) Patterns of nesting type showed no significant difference between genotypes (χ^2^=20.775, *p*>0.05). We coded repeated coding of ecological factors independently for this analysis due to statistical limitation.

**Fig. S1**

**
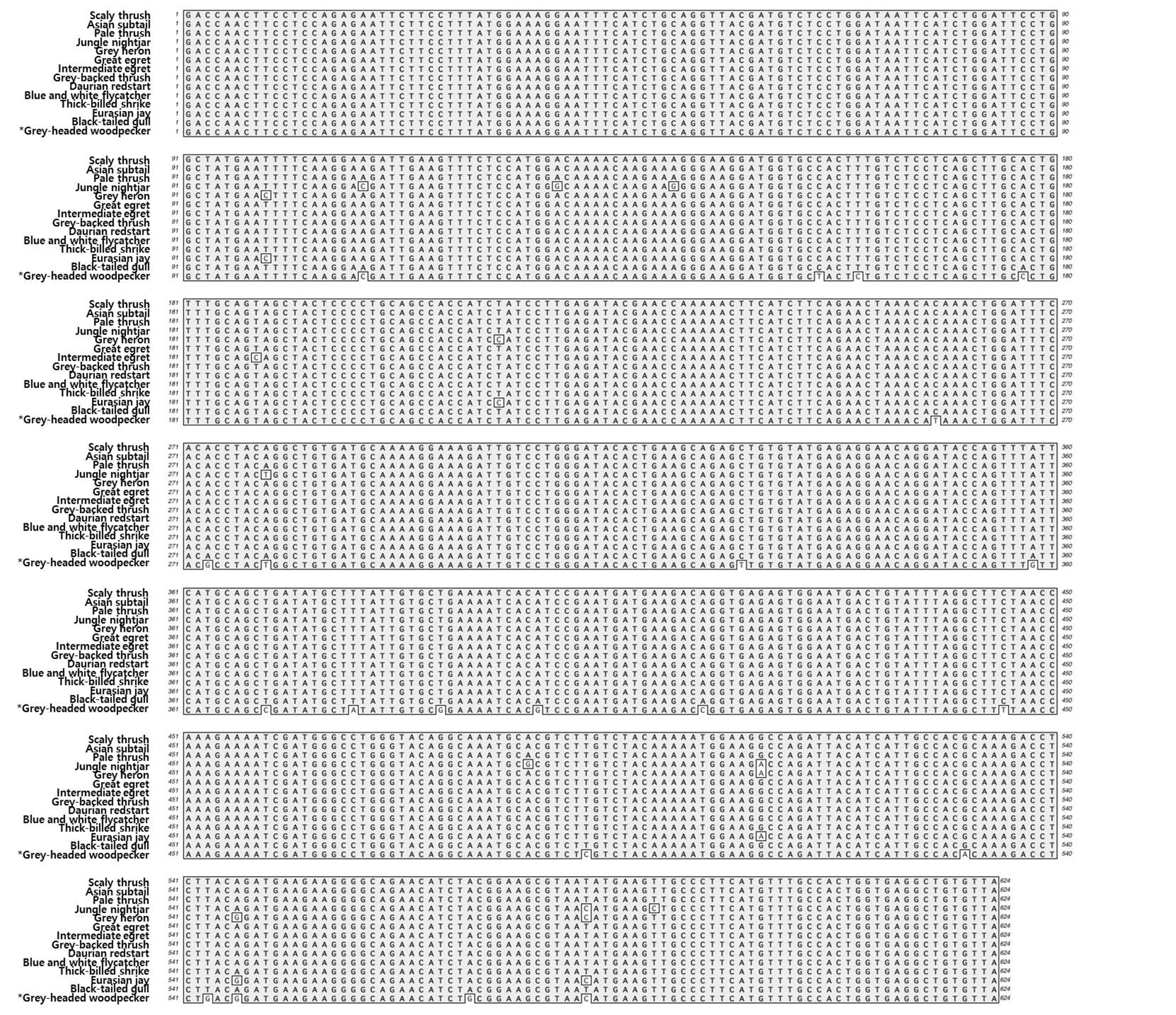
**

**Fig. S2**

**
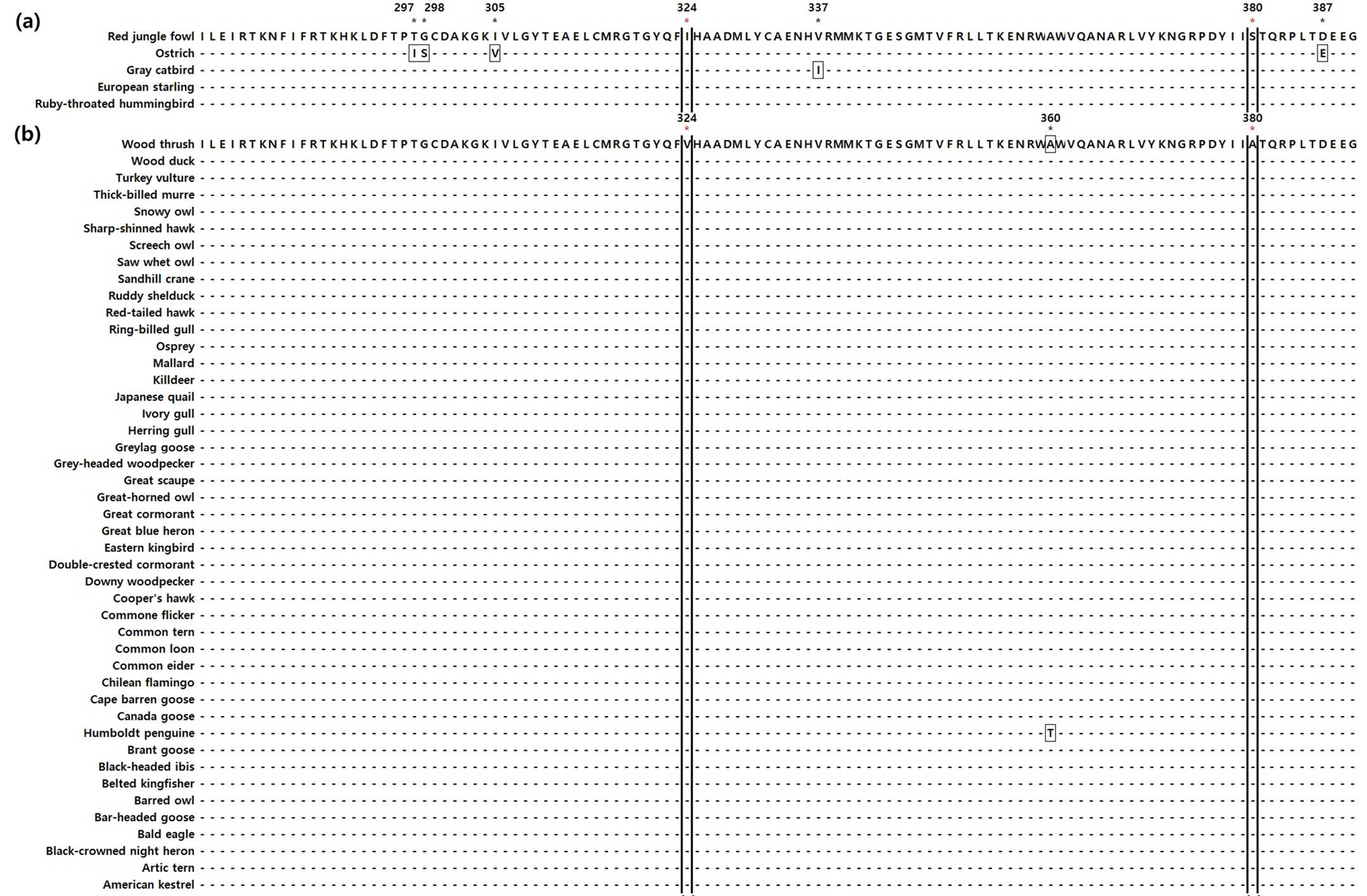
**

**Fig. S2 Continued**

**
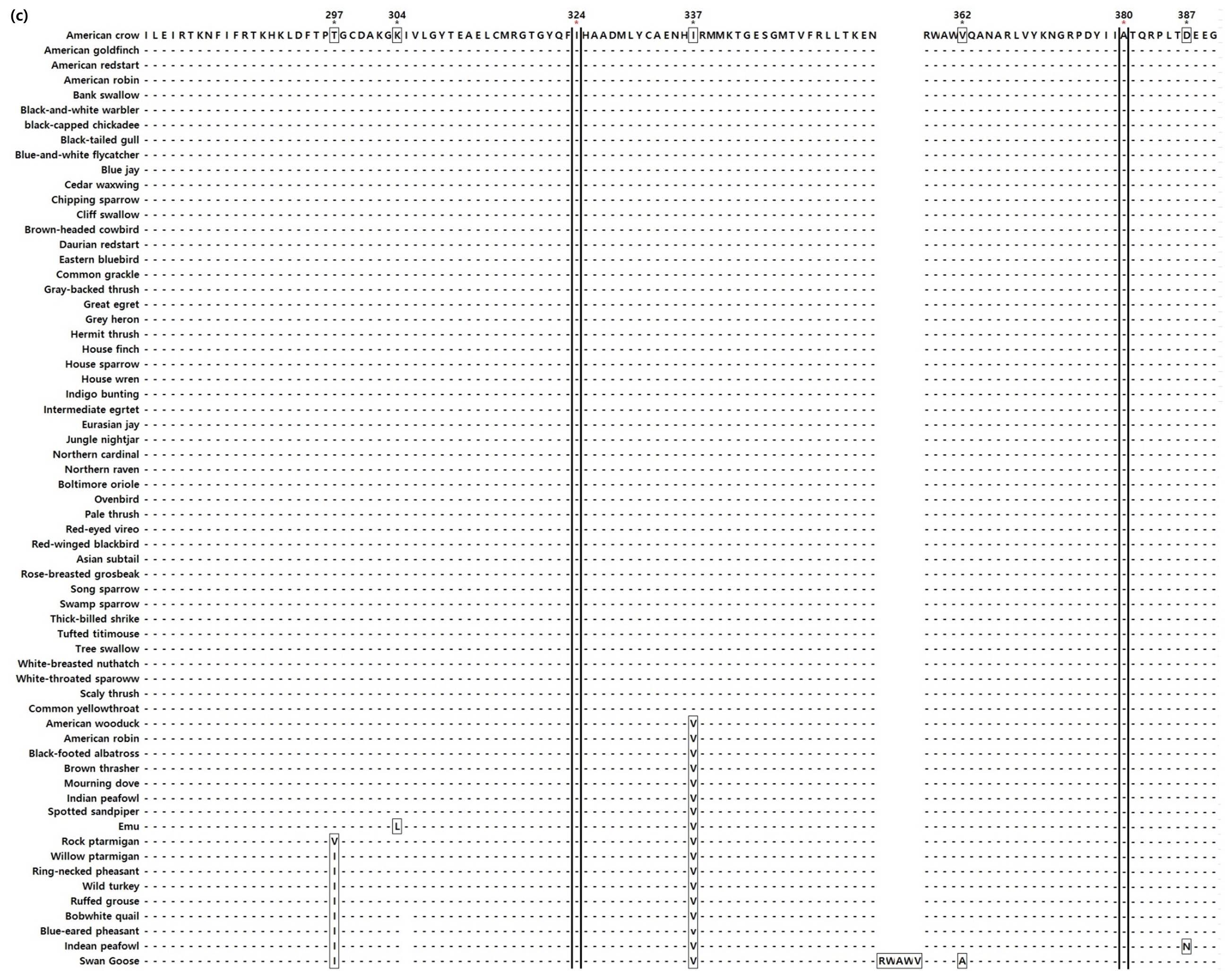
**

**Fig. S3**


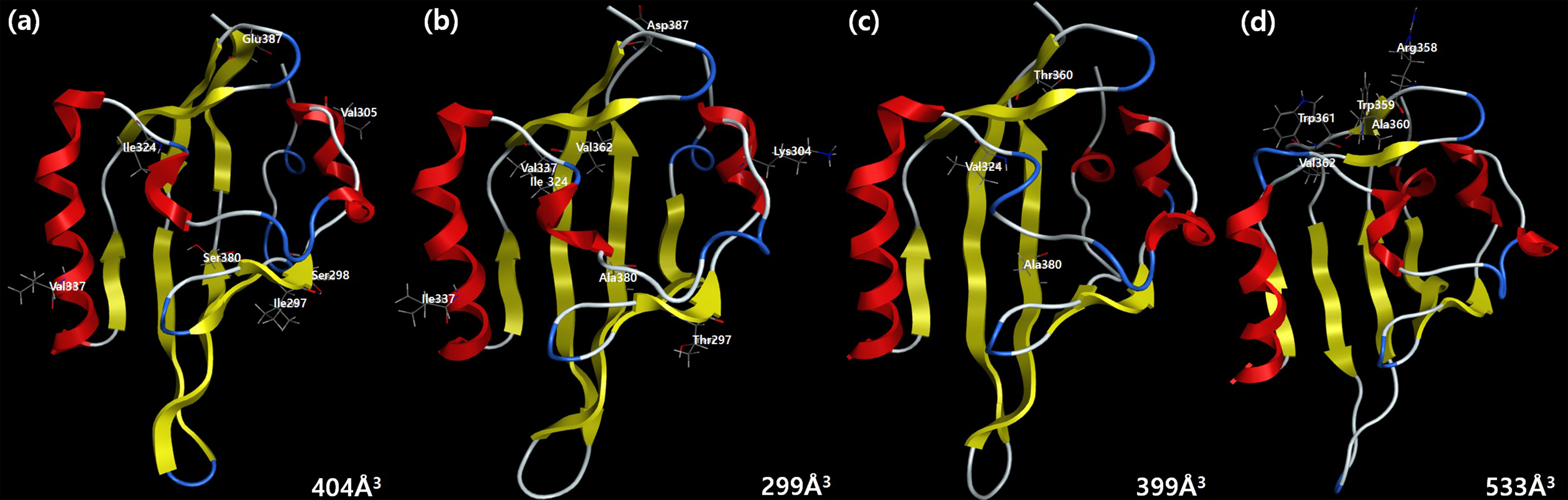


**Fig. S4**


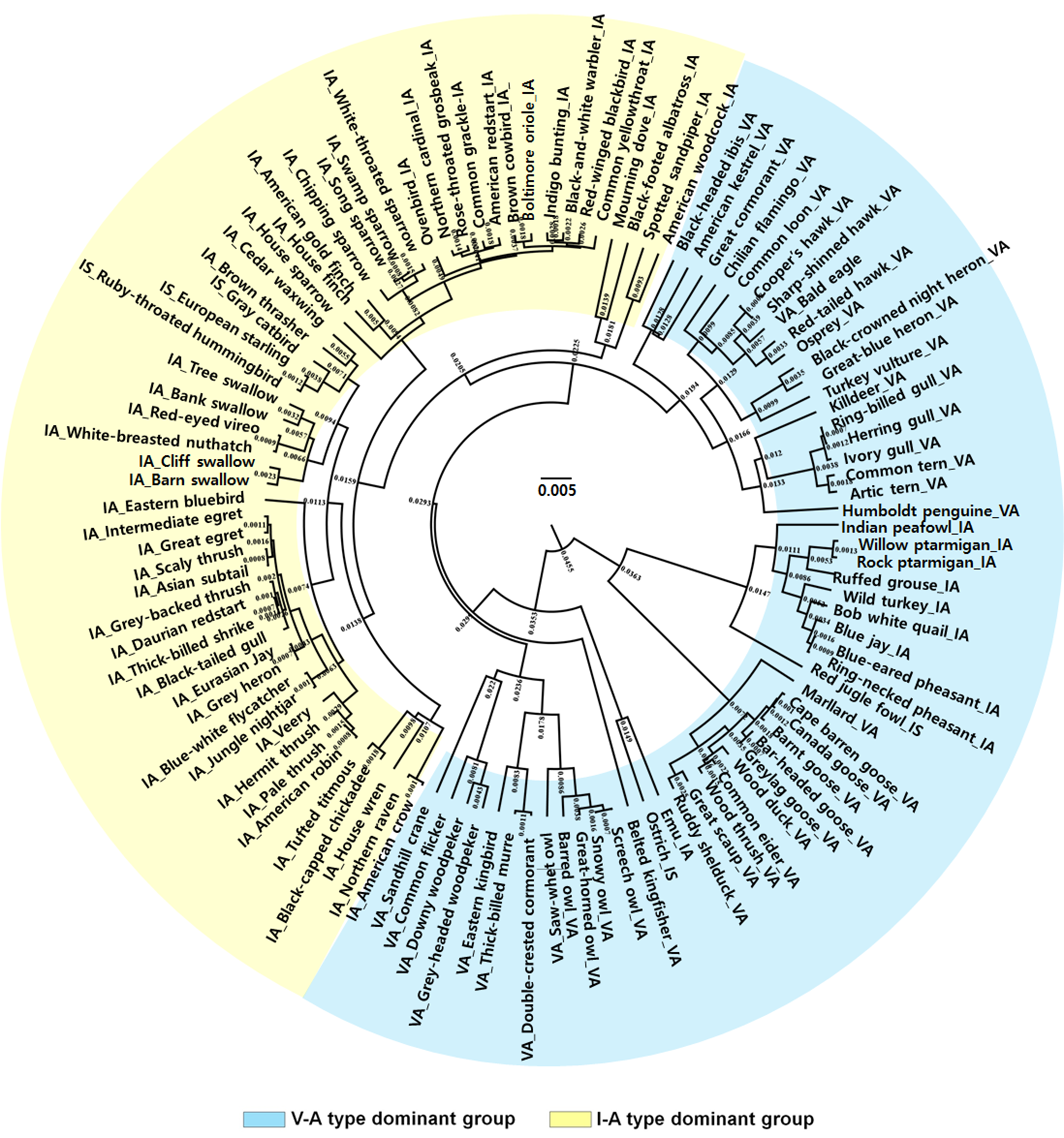


Fig. S5

**
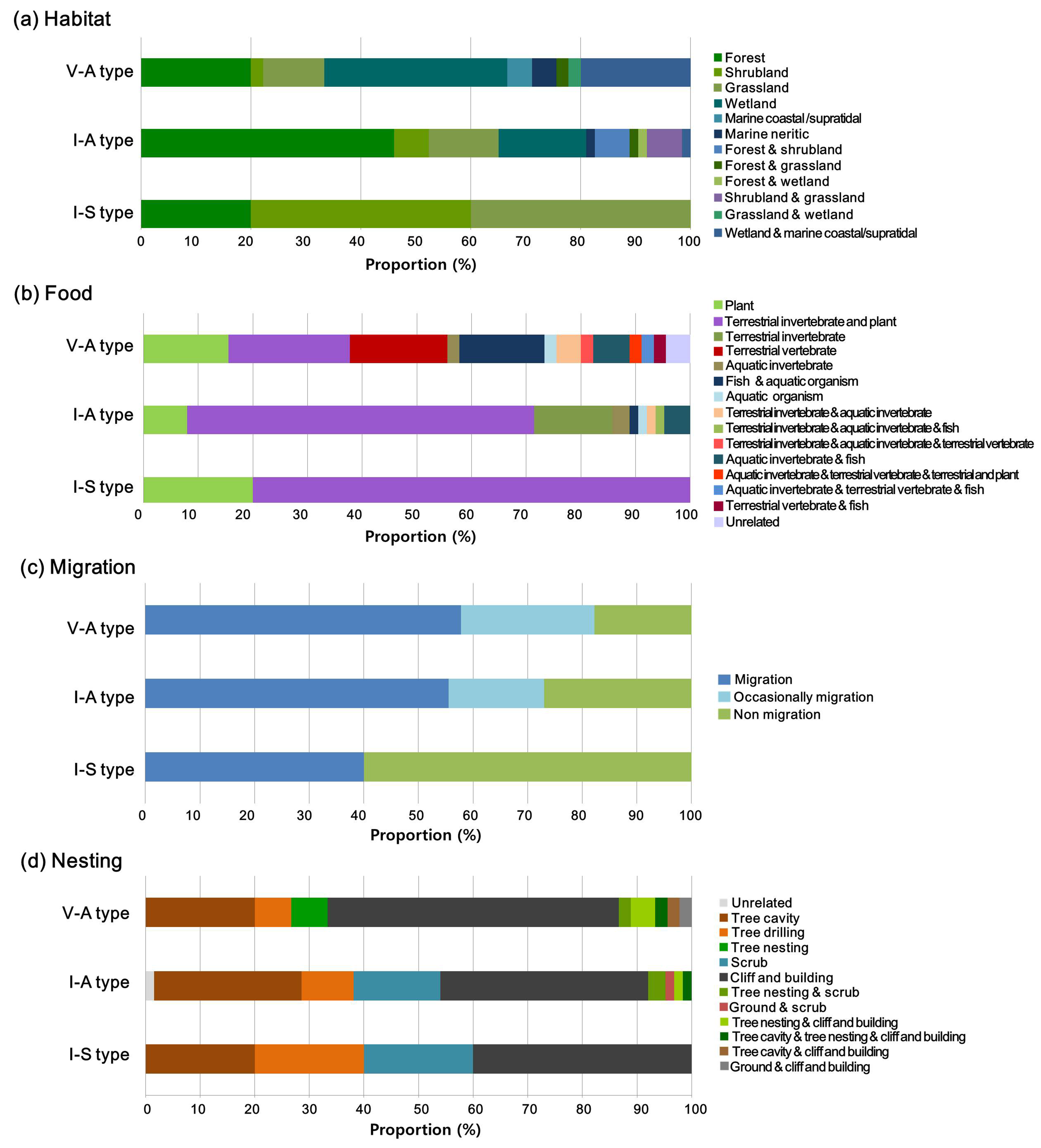
**
